# Influence of 17*α*-Methyltestosterone on Morphological Deformities and Pigmentation Development in Juvenile Japanese Eels, *Anguilla japonica*

**DOI:** 10.3390/ani14182684

**Published:** 2024-09-15

**Authors:** Ju-Ae Hwang, Jun Seong Park, Hae Seung Jeong, Seong Don Hwang

**Affiliations:** 1 Advanced Aquaculture Research Center, National Institute of Fisheries Science, Changwon 51688, Republic of Korea; hjuae1031@korea.kr (J.-A.H.); pjs5420939@gmail.com (J.S.P.); jhs0322@korea.kr (H.S.J.); 2 Division of Convergence on Marine Science, Korea Maritime and Ocean University, Busan 49112, Republic of Korea

**Keywords:** eel, 17*α*-methyltestosterone (MT), sex differentiation, abnormality, pigmentation 3

## Abstract

**Simple Summary:**

Artificial sex hormones such as 17*α*-methyltestosterone (MT) are used as useful tools for sex determination of major cultured fish species. However, the metamorphosis and embryology by MT treatment in Japanese eel *Anguilla japonica* remain largely unknown. In this study, MT induced male characteristics and morphological changes in juvenile eels, particularly a shortened snout length and pigmentation of the fin. Additionally, we successfully identified and demonstrated the involvement of specific genes associated with the determination of pigmentation, such as melanocortin 1 receptor (MC1R), tyrosinase (Tyr), and dopachrome tautomerase (DCT), following MT treatment. Our study presents the sex differentiation, deformities, and pigmentation development in the juvenile eels caused by MT treatment, providing basic knowledge for both growth and development studies analyzing sex steroids.

**Abstract:**

17*α*-methyltestosterone (MT) is a synthetic steroid used to induce masculinization when administered during the larval stage of fish. However, the side effects of MT on eel are still poorly understood and, in this study, we examined the various effects of MT on juvenile eel *A. japonica* (100.63 ± 8.56 mm total length (TL)). To further investigate growth and sex differentiation, juvenile eels (*n* = 1000) were exposed to 25 µg/g MT for 6 months. We analyzed growth-related factors, sex steroid hormones, skin pigmentation, and color-related gene expression. Through this study, we found a 90% sex conversion of juvenile eels to males using MT treatment. In the MT-treated eel group (285.97 ± 26.21 mm TL) where sexual maturity was induced, spermatogonia stages were observed in the gonads. In contrast, the control group (395.97 ± 27.72 mm TL) exhibited an 80% immaturity rate, with only 20% of the subjects that were rapidly developing displaying early oogonia. ELISA analysis results showed that the level of growth hormone, which is known to be secreted from spermatogonia, did not change as a result of MT treatment. We confirmed that MT delayed growth and caused morphological changes, particularly a shortened snout length and pigmentation of the fin. The total length, body weight, and snout length were considerably lower in the experimental group than in the control group. In addition, in histological analysis we also observed that some of the MT-treated group (5 out of 10 fish) showed liver atrophy and inflammation, and physiological analysis showed that the cortisol concentration increased in the MT-treated eels. Interestingly, we found that some pigment color-related genes, such as melanocortin 1 receptor (MC1R), tyrosinase (Tyr), and dopachrome tautomerase (DCT), were significantly overexpressed in the fins of MT-treated eels. These results suggest that the treatment of *A. japonica* larvae with MT induced masculinization but also causes growth side effects from the use of synthetic hormones.

## 1. Introduction

Japanese eel (*Anguilla japonica*) is one of 16 species of freshwater eel belonging to the *Anguilla* genus and widely distributed in East Asia [1]. Despite the success of artificial eel production in Japan [2], glass eel production depends on natural collection. Therefore, eels are highly valued in East Asian countries, and many studies on eel development and efficient aquaculture are underway [3,4]. Low egg and larval quality represent a persistent reproductive bottleneck for *Anguilla japonica* as well as for *Anguilla anguilla*, whose life cycles have been extensively studied [5,6,7]. Recently, various studies related to the production of artificial eel seeds have been reported, including the growth and morphological changes of hatched larvae [8], the production of recombinant eel luteinizing hormone [9] and the effects of recombinant eel growth hormone on larvae [10]. For the successful mass production of glass eels, settled management and sustainable supply of broodstock are important, because there is no gender distinction appearance; therefore, females and males are induced by artificial methods such as sex hormone (estradiol 17*β*, 17*α*-methyltestosterone, human chorionic gonadotropin) treatment. Environmental factors influence sex determination and gonadal development in fish [11,12]. As is well known, E2 (estradiol 17*β*) and MT (17*α*-methyltestosterone) are hormones used for feminization and masculinization, respectively [13,14,15].

Among sex steroids, MT is a synthetic steroid that has been widely used to masculinize species when administered during larval development [16,17]. However, MT has been used not only to induce masculinization [13] but also to improve egg quality and increase fecundity in European female eels [5]. It has also been reported to be involved in sex differentiation such as during the early ovary development of *A. japonica* [14]. Endocrine-disrupting chemicals (EDCs), also known as environmental hormones, widely exist in water and the atmosphere [5,16]. MT, an EDC, is a synthetic androgen that combines androgenic and estrogenic effects. It is usually detected in industrial and aquaculture wastewater [18,19].

Previous studies have been reviewed, including studies on the interference effects of MT on gonadal development and sex hormone synthesis, as well as related gene expression in fish, including eels (*A. japonica*) [20,21]. MT has been shown to disrupt the reproductive system and inhibit germ cell migration in *Gobiocypris rarus* [22]. Head-shape disparities and pollutant accumulation have been reported in wild-caught European eels [23]. Thyroid hormones cause musculoskeletal abnormalities in zebrafish during development [24]. As reported, EDCs such as MT may have other effects besides inducing sex determination.

However, no studies have demonstrated masculinization and other side effects of MT in the yellow eel *A. japonica*, with the exception of masculinizing effects on the developmental status of the eel [13]. Additionally, the disruptive effect of MT on eel growth (yellow to silver stage) remains unclear.

During the early life history of the genus *Anguilla,* they undergo several morphological changes [25,26]. The first metamorphosis is from larvae to glass eel; glass eels (48–55 mm) become elvers (50–70 mm) when they become pigmented, and then become yellow eels (71–74 mm) [26]. The second stage of metamorphosis is called “silvering”, as yellow eels become silver eels with a black-pigmented dorsum and a silvery shining belly [25,27]. Interestingly, despite the growing interest in the effects of MT on fish, including eels (*A. japonica*), particularly during the yellow stages, there is limited knowledge regarding its impact on eel deformities. In this study, we explored the multiple effects of MT on the body development and pigmentation of MT-treated eels in comparison to normal eels.

## 2. Materials and Methods

### 2.1. Fish and Breeding Environmental Control

Glass eels (57.8 ± 3.5 mm, 0.13 ± 0.02 g) were purchased from a fish farm located in Hampyeong-gun, Jeollanam-do, South Korea, to be used for our experiments. They were raised in tanks with aerated, recirculating fresh water at 25.0 ± 1.0 °C and dissolved oxygen (DO) of 8–9 mg/L for 1 month. After stabilization, yellow eel with total lengths of 100.63 ± 8.56 mm and body weights of 1.89 ± 0.32 g were used for our experiments; fish were divided into 2 groups for analyses: control (*n* = 1000) and MT-treated (N = 1000) groups. After being sorted into their respective groups, the experimental fish were placed in a 1.7 m width × 1.6 m length × 0.4 m height (water volume = 0.8 ton) fiber-reinforced plastic (FRP) tank in recirculating fresh water at 28 °C for 6 months. The eel stage was determined using morphological data previously published by Hatakeyama [26]. The eels in the control group were fed a commercial diet (Powder feed, SuHyupfeed, Republic of Korea), while the eels in the MT-treated group were fed a commercial diet containing MT (M0435, TCI) at a concentration of 25 µg/g diet for 6 months. Approximately 70% of the water was changed daily; the exchanged water was precipitated in a sedimentation tank containing activated carbon for 4 weeks and discarded after confirming the concentration of MT.

All procedures and investigations were approved by the National Institute of Fisheries Science (NIFS-2023-35) in Republic of Korea, and were performed in accordance with standard guiding principles.

### 2.2. Sampling Procedure and Measurement of Biological Indicators

Fish (N = 30) were investigated every month for 15 months after MT treatment. The overall length and weight of the eels were measured monthly at 30-day intervals, and the snout and prepectoral length were measured monthly from 6 months after treatment when measurement was possible (>20 cm).

GSI = (gonadal mass/body mass) × 100

HSI = (liver mass/body mass) × 100

Biological indicators included body weight (BW), total length (TL), liver weight, and gonad weight. The gonadosomatic index (GSI) and the hepatosomatic index (HSI) were calculated. To compare the external deformation of individual eels, the eyes, prepectoral length, and snout length were measured using Vernier calipers (CD-P30S; Mitutoyo Corp., Kawasaki, Japan) [28]. The color measurement of the eel skin (L*, a*, and b* values) [26] from around the dorsal section was performed using a colorimeter (TES 135A, TES electrical electronic Corp., Taipei, Taiwan) 6 months after treatment. The color values are defined as follows: L represents lightness, indicating the degree of lightness or darkness of a color, spanning a range from 0 (pure black) to 100 (diffuse white). Meanwhile, “a” represents the color axis from red to green, and “b” represents the color axis from yellow to blue.

### 2.3. Measuring Plasma Hormone Levels

The experimental eels (at 6 months after treatment) were anesthetized with MS-222 (Sigma, St. Louis, MO, USA), and blood (N = 10) was collected from the caudal vein using a 3 mL heparin-treated syringe. The collected blood was centrifuged at 2339× *g* at 4 °C for 15 min, and the resulting plasma supernatant was obtained and stored at −80 °C until analysis. The concentrations of 11-Ketotestosterone (11-KT), estradiol-17*β* (E2), growth hormone (GH), and cortisol in the plasma were determined using commercial ELISA kits (Catalog# MBS-269932, -9424416, -044656, -704055, Mybiosource, San Diego, CA, USA). Each sample, ranging from 50 μL to 100 μL, was tested, and all samples and standards were measured in three replicates.

### 2.4. Histological Analysis

After collecting blood, we dissected the gonads and liver from the eels (N = 10). Dissected tissues were fixed overnight in 10% neutral-buffered formalin and embedded in paraffin. Sections of 5 μm thickness were prepared, stained with hematoxylin and eosin, and examined under an optical microscope to assess the sex ratio and gonad development stage.

### 2.5. RNA Extraction and Quantitative Real-Time PCR Analysis

Fins of fish in each group (N = 10) were collected at 6 months after treatment for expression analysis of skin pigmentation genes. The fins were homogenized in TRIzol reagent (Invitrogen) using a motorized Kontes RNase-Free Pallet Pestle Grinder according to the manufacturer’s instructions. Total RNA purity was assessed using A260/280 and A260/230 NanoDrop UV spectrophotometry of both crude and column-purified RNA extracts. cDNA was synthesized using a first-strand cDNA synthesis kit (Roche) through reverse transcription. Real-time quantitative reverse transcription PCR (RT-qPCR) was used to analyze the expression of pigmentation-related genes. The primer sets used in this study were designed based on the NCBI GenBank (Table 1). The PCR cycling conditions were as follows: 40 cycles of denaturation at 95 °C for 10 s and annealing and extension at 60 °C for 45 s in a 7500 Real-Time PCR system (Applied Biosystems, Waltham, MA, USA). The expression of the genes was normalized to an endogenous reference, *β*-actin [29], and is presented as the difference between the target CT values and the *β*-actin CT values (ΔCT value). The comparison of gene expression between the groups and the calibrator was achieved by subtracting the calibrator ΔCT values from the target ΔCT values, resulting in a ΔΔCT value. Relative gene expression was calculated to determine fold difference (2^−ΔΔCT^).

### 2.6. Statistical Analysis

The data were subjected to *t*-test analysis using the SPSS statistical package (SPSS 24.0; SPSS Inc., Chicago, IL, USA). The significance between means was assessed using the SPSS program and *t*-tests, with significance levels set at *p* < 0.05, *p* < 0.005, and *p* < 0.001.

## 3. Results

### 3.1. Biological Indices

**Figure 1:**
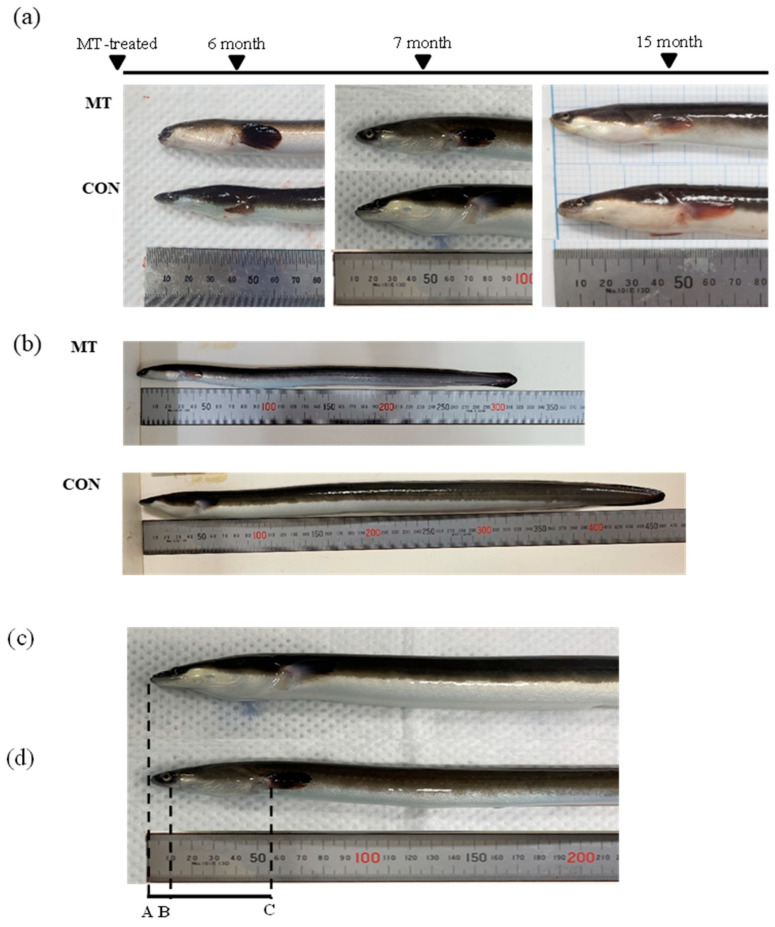
Morphologies of the studied *Anguilla japonica*. (**a**) Changes in the appearance of eels upon MT treatment. (**b**) MT-treated and control eels 7 months after treatment. (**c**,**d**) Control ((**c**); average length 404.97 ± 30.53 mm) vs. MT-treated ((**d**); average length 319.70 ± 30.00 mm) eels 7 months after treatment. A–B, snout length; B–C, prepectoral length.

**Figure 2:**
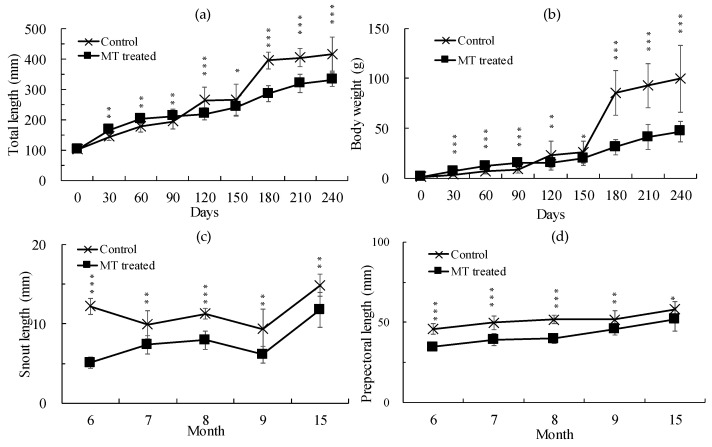
Time course of total length, body weight, snout length, and prepectoral length of the control and MT-treated *Anguilla japonica*. (**a**) Total length, (**b**) body weight, (**c**) snout length, and (**d**) prepectoral length. Data are expressed as means ± SD. Asterisks indicate significant differences (* *p* < 0.05, ** *p* < 0.01, *** *p* < 0.001) between the control and MT-treated eels (N = 30).

The effects of the MT hormone manifested in the very early stages of the yellow eel development. The first feature the eels demonstrated was a significant growth delay (*p* < 0.002, control: 254.52 ± 50.55 mm TL, 22.42 ± 8.36 g BW; MT: 218.77 ± 19.82 mm TL, 15.14 ± 4.81 g BW) after 120 days of MT treatment (Figure 1b and Figure 2a,b). This phenomenon persisted up to 210 days (7 months) after MT treatment and remained similar even at 15 months, which is 9 months after the discontinuation of MT. Interestingly, up to 90 days after MT treatment, the growth of the MT-treated eels was markedly faster than that of the control eels (Figure 2a,b). However, after that point, growth in the MT-treated group considerably decreased and remained lower until 15 months (Figure 2a,b; Table 2).

Second, the MT-treated eels showed abnormal development in snout, prepectoral, and eye lengths compared to the control eels (Figure 2b,c; Table 2). This phenomenon was observed from the onset of the MT treatment, and after 6 months of treatment, markedly shortened snout and prepectoral lengths were evident in the MT-treated eels. When comparing eye length, it became apparent that in the MT-treated group, it began to lengthen from 6 months onwards compared to the control group. However, a significant increase was noted 15 months after MT treatment (*p* < 0.05, Table 2).

Moreover, the MT treatment group exhibited pigmentation in the pectoral and caudal fins in contrast to the control group. Pigmentation initiation was observed as early as the first month of MT treatment and persisted until 6 months of MT treatment (Figure 1a). It started as the darkest observed shade and gradually lightened (Table 2). After 6 months of MT treatment, when measured with a colorimeter and compared with the control group, lower L values indicated darker pigmentation, and these were considerably lower in the MT group. Additionally, the a and b values were considerably lower and higher, respectively, in the MT group (Table 3).

### 3.2. Histological Analysis

**Figure 3:**
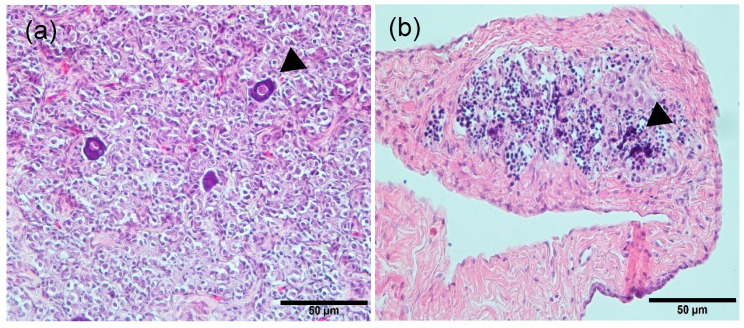

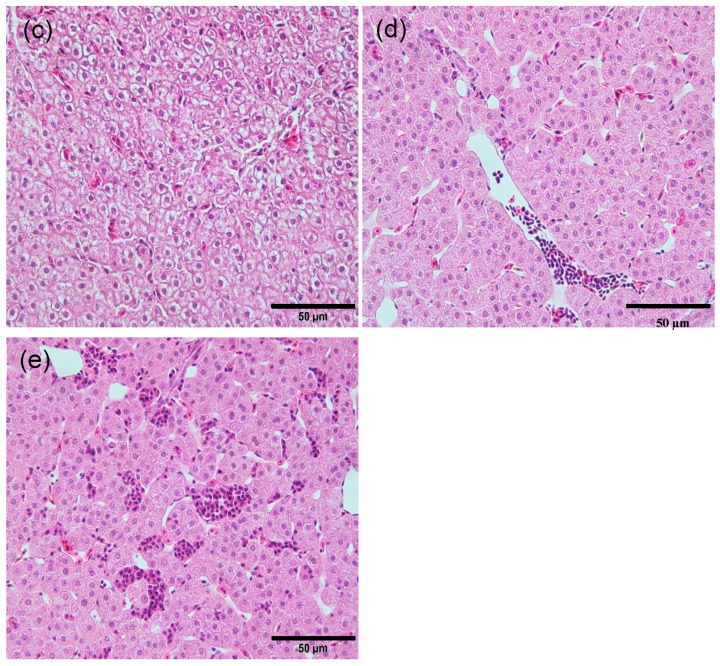
Hematoxylin–eosin (H-E) staining of morphological gonads and liver in *Anguilla japonica* 6 months after MT treatment. (**a**) Control gonad (ovary of a 41.7 cm long eel, stage 0, oogonia). (**b**) MT-treated gonad (testis of a 28.3 cm long eel, stage 0, spermatogonia). (**c**) Liver showing a normal structure in the control group. (**d**) Liver showing atrophy and (**e**) the appearance of immature red blood cells in the MT-treated group. Arrowheads represent notches in the oogonia (**a**) and spermatogonia (**b**). Scale bar, 50 µm.

Sex ratio analysis through histological examination revealed a male prevalence of 90% at 6 months (285.97 ± 26.21 mm TL, 31.16 ± 7.57 g BW). In contrast, the control group (395.97 ± 27.72 mm TL, 85.24 ± 22.39 g BW) mostly comprised immature individuals, with only 2 out of 10 showing sexualization (Figure 3, Table 4). Among the control eels, those that had completed sex determination and were sexualized as females met the criteria of a total length of 400 mm or more and a body weight of 90 g or more. They exhibited oogonia in an immature state (stage 0) (Figure 3a). Eels induced into males via MT treatment also displayed stage 0 spermatogonia (Figure 3b), and 76% of them had a length of less than 300 mm. A histological examination of the liver revealed liver atrophy in 50% (five out of ten fish) and the presence of immature erythrocytes within the liver parenchyma in some individuals in the MT-treated group (Figure 3d,e).

### 3.3. Serum 11-KT, E_2_, GH, and Cortisol Concentrations

Although the difference was not significant, E2 and GH levels were slightly increased after MT treatment in serum. 11-KT was not detected in either the control or MT-treated group. In the case of cortisol, a health and stress index, the MT experimental group showed a significant increase (*p* < 0.01) compared with the control group (Table 4).

### 3.4. Skin Pigmentation Gene Expression

**Figure 4:**
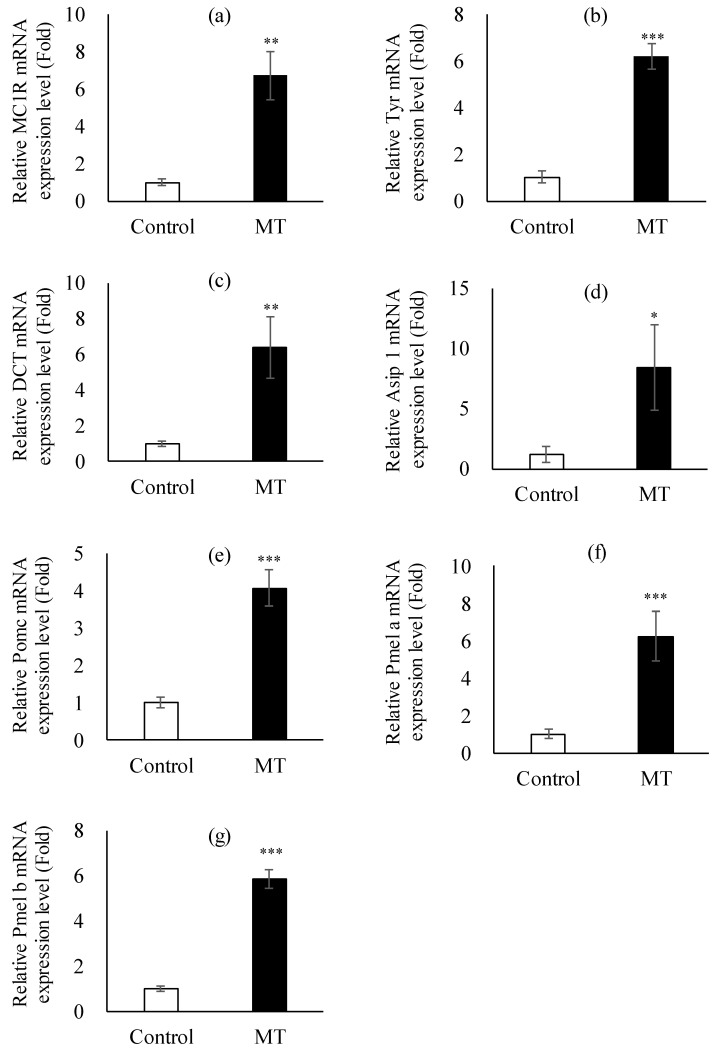
Expression of skin pigmentation genes in the fins. (**a**) Melanocortin 1 receptor (MC1R), (**b**) tyrosinase (Tyr), (**c**) dopachrome tautomerase (DCT), (**d**) agouti-signaling protein (Asip), (**e**) pro-opiomelanocortin (Pomc), (**f**) premelanosome protein (Pmel a), and (**g**) premelanosome protein (Pmel b). A quantitative real-time PCR analysis was performed with equal amounts of total RNA isolated from fins of eels. β-actin was used as an internal control. Asterisks indicate significant differences (* *p* < 0.05, ** *p* < 0.01, *** *p* < 0.001) between the control (N = 10) and MT-treated groups (N = 10).

The expression of all skin-pigmentation-related genes was considerably higher in the MT treatment group than in the control group (Figure 4). Melanocortin 1 receptor (MC1R), tyrosinase (Tyr), and dopachrome tautomerase (DCT) were significantly (*p* < 0.01, *p* < 0.001) higher in the MT-treated group (6.66-fold, 6.05-fold, and 6.35-fold, respectively) compared with the control group (Figure 4a–c). Additionally, other pigment-related genes were upregulated in the MT-treated eels compared with those in the control group (Figure 4d–g).

## 4. Discussion

This study found multiple effects on MT-treated eels, including masculinization, growth delay, snout and prepectoral length abnormalities, and pigmentation. For the mass production of farmed fish, such as tilapia [30], artificial sexual maturation or sex reassignment methods such as hormone treatment are used. MT is an important hormone used for masculinization. It has been reported that MT has a masculinizing effect on many fish [14,17,31] and reptiles, such as American alligators [32]. In elver eels (16.6 ± 0.7 cm), testicular maturation induced by MT treatment at a concentration of 100 µg/g resulted in the confirmation of 100% spermiogenesis within 45 days of treatment [13]. In this study, a 90% masculinization rate was achieved in eels (218.77 ± 19.82 mm) treated with a low concentration of 25 µg/g after 6 months of treatment (Figure 3b, Table 4). The concentration was chosen because when treated with 40 µg/g and 25 µg/g in our preliminary experiments, the 40 µg/g group showed severe hepatomegaly and liver congestion that even caused abnormal food intake.

ELISA analysis (Table 4) revealed that 11-KT, a hormone known to be associated with testis development, was not detected. This is consistent with a report stating that detection of 11-KT in the blood is incompletely confirmed even in adult silver eel [29]. GH and E2 levels seemed to increase slightly in the MT-treated group, but not significantly. E2 decreased (<0.1 ng/mL) after eels were orally administered 5 mg MT in a previous study [14], but also not significantly, like in our study. There are reports that MT is involved in oocyte maturation [14], but there does not seem to be a direct relationship with E2. It is worth noting that GH has been previously reported to stimulate spermatogonial proliferation in the testis of eels [32]. MT increased oil droplets and related early ovary development in adult migrating eels (*A. japonica*, *A. anguilla*) [33,34]. However, in this study, there were no significant differences in E2 and GH between the two groups. Androgens are likely important during spermatogenesis and oogenesis. Moreover, eel gonadal development is a complex process, and certain stages of sex development become evident over time. This aligns with previous research where MT’s effects were primarily studied in elvers, focusing on the emergence of spermatogenesis on the day of MT treatment [13]. Although the relationship between the sex-related hormones caused by MT treatment in yellow eels was not revealed, we believe that it will be necessary to investigate the expression of genes related to spermatogenesis and the development of gonads in future research.

MT has been reported to not only induce sex differentiation but also cause deformities [22,35,36]. In *Pseudorasbora parva*, MT inhibits gonadal development and causes growth delays [35]. Our study also showed the already known masculinization effects and growth delays caused by MT (Figure 1, Table 2), as have previous studies. Additionally, in *G. rarus*, MT is known to cause abnormal gonad development by affecting the expression of key genes in the PI3K/AkO3a and hormone production pathways (gnrh3, gnrhr1, and cyp19a1b) [28]. Other studies have reported that MT treatment affects the pigmentation of red cichlids [31]. In both ornamental fish and eels, MT causes pigmentation; in our study, this manifested as blackening.

MT has been shown to disrupt the reproductive system and inhibit germ cell migration in *G. rarus* [22]. Head-shape disparities and pollutant accumulation have been reported in wild-caught European eels in Belgium [23]. Thyroid hormones cause musculoskeletal abnormalities in zebrafish during development [24]. In this study, significant reductions in snout length, prepectoral length, and pigmentation were observed when compared to the control group (*p* < 0.001, Figure 1a and Figure 2c,d, Table 2). In addition, after 3 months of treatment, growth was noticeably delayed compared to the control group (Figure 2a,b). Similarly to the reported disruptive effect of MT in other fish, our results also showed that MT can inhibit the growth and normal development of yellow eel.

In adult *Gambusia holbrooki,* MT stimulates development of secondary sexual characteristics, i.e., the formation of testicular tissue and changes in fins [37]. This is similar to observations in zebrafish [24], in which thyroid hormones cause musculoskeletal abnormalities during development, suggesting that skeletal or developmental abnormalities are affected by MT treatment. A group targeting male silver eels (body weight 80–115 g) also reported darkening of the pectoral and caudal fins due to MT injections [38]. These pigmentation results are consistent with our results, except that the treatments were performed at different growth stages. Another group treated elver eels with MT in a manner similar to ours, although their treatment concentration and duration were notably lower and shorter than ours. Furthermore, their study exclusively focused on the induction of male sex differentiation via MT [13].

Pigmentation serves as an indicator of developmental changes during the transition from the yellow to the silver eel stage, a critical process in the sexual maturation and differentiation of eels [27]. In our study, the experimental group treated with MT displayed pigmentation one month after MT treatment. Remarkably, these symptoms persisted throughout the MT treatment and continued for up to one month after treatment cessation (Figure 1). It is worth noting that the average total length and weight of naturally occurring stage s1 silver eels (4–6 years old) are reported to be 239 ± 74 mm and 55.4 ± 5.1 g for males [27].

At the 6-month mark of MT treatment, the experimental group exhibited an average total length and weight of 285.97 ± 26.21 mm and 31.16 ± 7.57 g, respectively, displaying a growth pattern similar to that of the natural stage s1 group. In this study, black pigmentation similar to silvering was confirmed in the group artificially treated with MT. In contrast, the control group had an average total length and weight of 395.97 ± 27.72 mm and 85.24 ± 22.39 g, respectively. While the male eels in the control group corresponded to stages Y1–Y2, they remained in an undifferentiated state of sexual maturity, and pigmentation was not observed [27].

Several genes play crucial roles in pigmentation, including MC1R, Tyr, and DCT [39]. However, there have been no reports to date regarding the expression of these genes during the transition from yellow to silvering in eels. In our study, we investigated the expression of genes related to pigmentation induced by MT treatment. The RT-PCR results revealed a significant increase in the expression of most pigment-related genes, including Tyr and MC1R, in the MT-treated group (Figure 4). In addition, the colorimeter measurement results showed that the L value was considerably lower in the MT group (Table 4). In some fish, including carp, a relationship between pigmentation caused by stress and increased MCR gene expression has been reported [38]. However, no studies have conducted a genetic analysis of pigmentation in eels. According to our results, the pigmentation induced by MT appears to follow a signaling pathway involving the overexpression of MC1R, tyrosine, and DCT. This is consistent with the results of an association between the MCR1 gene and black pigmentation in koi and carp [39,40]. There will be a need to investigate the relationship between sexual maturity and pigmentation-related genes in individuals in the sexual maturity stage through future research.

MT not only ed to growth delay and developmental abnormalities in eels but also induced hepatotoxicity in 50% of the eels (Figure 3d), as observed upon histological analysis. Additionally, ELISAs confirmed that cortisol secretion in the blood was considerably increased (Table 4). The liver plays crucial roles in energy metabolism and hormone synthesis, including insulin-like growth factors and thrombopoietin, and is a subject of investigation in veterinary toxicological studies among other organs [41,42]. Similar to our findings, previous research on Nile tilapia reported liver alterations in response to MT treatment [30]. However, contrasting results were observed in silver eels, where no liver damage was reported despite hormone treatment [43]. These disparities can likely be attributed to differences in the age and growth stage of the eels when subjected to hormonal treatment. Future studies should explore the effects of hormones on eels considering their age and sexual maturity stages.

In this study, we conducted a pioneering investigation into the impact of the MT hormone on growth and abnormal development in yellow eels (*A. japonica*) and proposed potential deformity effects caused by MT. Therefore, these results may provide important information on artificial seed production of eel.

## 5. Conclusions

This study was performed to determine the effects on growth and abnormal development during the yellow eel stage caused by MT treatment. When applied to yellow eels, analysis of MT treatment has revealed various abnormalities, encompassing masculinization, growth retardation, and cranial deformities. In our MT-treated experimental group, histological examination confirmed 90% masculinization, alongside an increase in GH associated with spermatogonia development. MT treatment not only influenced sex differentiation but also contributed to growth retardation, shortened snout length, and reduced dorsal origin distance. Furthermore, MT treatment induced hepatotoxicity and elevated cortisol secretion in the eels. Pigmentation serves as a crucial indicator of silvering in eels during their transition from the yellow stage to the silver stage. MT treatment induced pigmentation resembling the natural silvering process, and this melanin pigmentation was associated with the overexpression of the *MCR1*, *TYR*, and *Asip1* genes. However, the exact relationship between this phenomenon and the artificial induction of male sex differentiation and silvering remains unclear.

Future research endeavors should focus on elucidating the connection between pigmentation and related genes during the artificial induction of sex differentiation and the silvering stages.

## Figures and Tables

**Table 1:** List of primers used in this study.

Genes	Forward (′5 → ′3)	Reverse (′5 → ′3)	Amplicon Size (bp)
**Melanocortin 1** **receptor** **(MCR1)**	TGGAGCACAATCCTTTGATGTA	TCGTGCGGGATCATAACTTG	148
**Tyrosinase** **(Tyr)**	CGGTGCTAATTGTGCTGAAAG	TCTTTGCCAGGTTCAAGTAAGA	119
**D** **opachrome** **tautomerase** **(DCT)**	TTTCCGGGAGAGAACCATTAAC	GAAGGTGGAGTTGGTGAAGTAG	138
**A** **gouti signaling protein 1** **(Asip 1)**	ACTGCCAAGTGTTTGCTTTG	ACTGCTGGAAGACGCATTTA	131
**Proopiomelanocortin** **(Pomc)**	TCACCCTCCAGACCAAGAA	AGCCACCGTAGCGTTTG	124
**P** **remelanosome** **protein a** **(Pmel a)**	TCTACCCAGACAGAAACTCCTC	CGTCTTCCACACGTACACATAG	118
**P** **remelanosome** **protein b** **(Pmel b)**	GGGCATTGAGAATGTGGAGATA	CAGGCTTCCTTGACAGATGAT	100
**β-actin**	AATCCACGAGACCACCTTCAACT	TGATCTCTTTCTGCATTCTGTCG	134

**Table 2:** Morphometric measurements of cultured *Anguilla japonica* for 6 and 15 months after treatment.

	6 Months		15 Months	
	Control	MT Treated	Control	MT Treated
TL (mm)	395.97 ± 27.72	285.97 ± 26.21 ***	462.40 ± 36.2	404.7 ± 43.5 ***
BW (g)	85.24 ± 22.39	31.16 ± 7.57 ***	131.00 ± 32.47	87.00 ± 30.57 ***
SL (mm)	12.22 ± 0.99	5.10 ± 0.74 ***	14.86 ± 1.39	11.78 ± 2.24 **
PL (mm)	45.80 ± 3.36	34.70 ± 1.64 ***	58.17 ± 4.74	51.92 ± 7.17 *
EL (mm)	3.71 ± 0.54	4.12 ± 0.30 ^ns^	4.96 ± 0.99	4.18 ± 0.43 *

Significant difference (* *p* < 0.05, ** *p* < 0.01, *** *p* < 0.001) between the control eels and MT-treated eels; TL (total length), BW (body weight), SL (snout length), PL (prepectoral length), EL (eye length) and ns, not significant.

**Table 3:** Color parameters measured in skin of eels during the experimental period 6 months after treatment.

Color Parameters	Control	MT-Treated
L*	9.38 ± 1.02	8.07 ± 0.96 **
a*	3.87 ± 1.11	3.65 ± 0.09 ^ns^
b*	8.30 ± 0.81	9.55 ± 0.43 ***

Significant differences (** *p* < 0.01, *** *p* < 0.001) were observed between the control and MT-treated eels. ns, not significant.

**Table 4:** The sex ratio and sex-related hormone levels of the eels 6 months after treatment.

	Control	MT Treated
Sex ratio % (female/male)	20:ND	10:90
Estradiol-17*β* (pg/mL)	76.4 ± 33.80 ^ns^	140.3 ± 177.4 ^ns^
11-kt (pg/mL)	ND	ND
GH (pg/mL)	12.9 ± 6.20 ^ns^	14.6 ± 4.1 ^ns^
Cortisol (ng/mL)	7.7 ± 1.51 ^ns^	15.2 ± 3.77 **

Significant difference (** *p* < 0.01) between control and MT-treated eels; ns, not significant; ND, not detected.

## Data Availability

The data presented in this study are available on request from the corresponding author.
